# Stimuli-Responsive Self-Assembly of Poly(2-(Dimethylamino)ethyl Methacrylate-co-(oligo ethylene glycol)methacrylate) Random Copolymers and Their Modified Derivatives

**DOI:** 10.3390/polym15061519

**Published:** 2023-03-19

**Authors:** Antiopi Vardaxi, Stergios Pispas

**Affiliations:** 1 Theoretical and Physical Chemistry Institute, National Hellenic Research Foundation, 48 Vassileos Constantinou Avenue, 11635 Athens, Greece; 2 Department of Chemistry, National and Kapodistrian University of Athens (NKUA), 15784 Athens, Greece

**Keywords:** random copolymers, polyelectrolytes, quaternization, pH-response, temperature-responsive behavior, ionic strength, intra-molecular self-folding, inter-molecular self-assembly, unimer, multichain aggregates

## Abstract

In this work, the synthesis and the stimuli-responsive self-assembly behavior of novel double-hydrophilic poly(2-(dimethylamino)ethyl methacrylate-co-(oligo ethylene glycol)methacrylate) random copolymers and their chemically modified derivatives are presented. The synthesis of P(DMAEMA-co-OEGMA) copolymers of different DMAEMA mass compositions was successfully conducted through RAFT polymerization, further followed by the hydrophilic/hydrophobic quaternization with methyl iodide (CH_3_I), 1-iodohexane (C_6_H_13_I), and 1-iodododecane (C_12_H_25_I). The tertiary and quaternary amines are randomly arranged within the DMAEMA segment, responding thus to pH, temperature, and salt alterations in aqueous solutions. Light scattering techniques elucidated the intramolecular self-folding and intermolecular self-assembly of polymer chains of P(DMAEMA-co-OEGMA) copolymers upon exposure to different pHs and temperatures. Q(P(DMAEMA-co-OEGMA)) cationic polyelectrolytes demonstrated moderate response to pH, temperature, and ionic strength as a result of the permanent hydrophilic/hydrophobic profile, closely connected with the attached alkyl chains and the quaternization degree. Moreover, fluorescence spectroscopy measurements confirmed the internal micropolarity and the picture of the aggregate inner structure.

## 1. Introduction

Self-assembled copolymers have been investigated for many years owing to their assortment of morphologies and applications in different fields of interest, especially in biomedical ones, including drug delivery systems, tissue engineering, and theranostics [1,2,3,4]. The formation of unprecedented nanosized structures driven by the self-assembly forces when inserted into aqueous media enables copolymers to be widely utilized in nanotechnology [5]. This behavior is closely associated with the molecular mass, composition, sequence distribution, and macromolecular architecture of the copolymers and the selected comonomers [6]. 

Copolymers can be broadly divided into block and random (statistical) copolymers. Block copolymers are constituted of well-arranged sequences of similar monomers in the form of blocks. The exhibition of extraordinary self-assembly behavior and the prediction of final precise/periodic structures through the modulation of molecular features, such as molecular weight, composition, and block lengths, have contributed to the extended scientific focus on such synthetic macromolecules [7,8]. On the other hand, random copolymers are composed of monomers randomly arranged into the forming polymeric chain where the likelihood of locating a given unit at a certain position of the chain is not reliant on the nature of adjoining units at this site [9]. The preparation of random copolymers is facile yet fruitful, as it can be accomplished in a single-step copolymerization of two (or more) monomers. However, the difficulty of creating well-structured morphologies with narrow dispersity using random copolymers with wide molecular distribution has hampered intense self-assembly studies [10]. The versatile living/controlled radical polymerization techniques brought to prominence the synthesis of self-assembled random copolymers of narrow molecular weight dispersity and the ability of their industrial-scale production, two aspects that contribute to the emerging scientific interest in random copolymers [11,12]. Once amphiphilic random copolymers are inserted into aqueous solutions, they tend to induce intramolecularly self-folding into unimolecular nanosized micelle-like aggregates due to the segregation of the hydrophobic monomeric units as opposed to analogous block copolymers, which self-assemble yielding spherical multichain core-shell micelles [13,14]. The hydrophobic-to-hydrophilic ratio (also referred to as hydrophilic–lipophilic balance (HLB)) and the chain length of amphiphilic random copolymers constitute the most crucial parameters for their self-organization [10,15,16]. When the hydrophobic content is low, single-chain formations, namely unimers, arise through intramolecular self-folding, in which the hydrophobic inner domain is comprised of the hydrophobic monomers. When the hydrophobic content is increased, multichain aggregates driven by intermolecular interactions are formed [13,17]. The composition and chain length of the random copolymer system (composition-dependent threshold degree of polymerization, DP_th_), below which the copolymers self-assemble intermolecularly into multichain nanostructures, define the transition between unimolecular and multimolecular aggregates [17,18]. In a similar way to intramolecular and/or intermolecular self-assembly in water, it is noteworthy to be mentioned that proteins/enzymes can yield inherent tertiary and quaternary structures since they possess distinct primary structures (molecular weight and monomer sequence), further resulting in structures stabilized by hydrogen bonding and covalent bonding. Hence, random copolymers synthesized with controlled radical polymerization imitate the folding/aggregation of proteins and can be utilized as simple models of protein behavior in aqueous solutions [19].

Stimuli-responsive polymers, which are also referred to as smart polymers, have emerged as the key constituents in the contemporary generation of supramolecular nanoformulations with versatile structures, owing to their ability for structural and physicochemical alterations when they are triggered by a stimulus, either exogenous, such as temperature variations and magnetic field, or endogenous like pH, redox, enzymes, and hypoxia, thereby appointing them as eligible multifunctional nanocarriers for drug delivery systems [20,21,22]. Amongst the multitude of responses, the one to temperature was presented as the stimuli of preference in the majority of studies, also due to similarities with the thermal denaturation of proteins in solution. Some stimuli-responsive polymers display lower critical solution temperature (LCST), i.e., the lowest temperature at which chain conformation transition and phase separation from solution occur. Hence, beyond the LCST, an entropy process leads to phase separation, whilst beneath the LCST, both polymer chains and molecules of solvent are homogenously mixed [23]. One of the well-studied thermoresponsive polymers is poly(N-isopropyl acrylamide) (PNIPAm), in which the phase transition from a hydrophilic state to a hydrophobic one takes place at 32 °C in water; a temperature near the normal human body one (37 °C) [24]. Furthermore, polymers bearing acidic (e.g., -COOH) or basic (e.g., NH_2_-) functional groups are prone to ionization according to solution pH value; acidic polymers are ionized at high pH values, as opposed to basic ones, which are ionized at low pH [25]. Some polymers are triggered by more than one stimulus, such as both pH and temperature. Poly(2-(dimethylamino)ethyl methacrylate) (PDMAEMA) is such a dual temperature and pH-responsive polymer that it receives considerable attention. PDMAEMA is a weak cationic polyelectrolyte with pK_a_ ca. 7.4, in which dimethylamino groups are partially protonated under physiological conditions, whilst the amine side groups can be fully (de)protonated when the pH is above or below the pK_a_, respectively [26,27]. PDMAEMA can also be selectively chemically modified utilizing conventional alkyl halides, thereby introducing permanent cationic charges along the polymer chain and a high hydrophilic profile and transforming it into a strong polyelectrolyte [28]. The transition phase from a hydrophilic to a more hydrophobic state is above LCST (40–50 °C) since the high temperature provokes the fracture of hydrogen bonds between the polymer chain and the water molecules [29]. The LCST can be tuned either by varying the protonation degree or the quaternization degree of tertiary amine groups or even by the chain length of alkylating pendant agents, producing thus nanostructures for biomedical applications [30]. Even though PDMAEMA displays less cytotoxicity than other cationic polymers, its lessened colloidal stability restricts its utilization in in vivo applications [31]. An efficient method to ameliorate these limitations without sacrificing the transfection efficiency, originating from the cationic nature of PDMAEMA, includes the modification/copolymerization with hydrophilic poly(ethylene glycol) (PEG) units (PEGylation process) or with the graft-like analogous polymers, namely poly(oligo ethylene glycol)methacrylate) (POEGMA) [31,32]. POEGMA is a non-ionic oligomer with a hydrophilic profile. It comprises a hydrophobic carbon–carbon backbone and grafted hydrophilic multiple oligo(ethylene glycol) side chains. The number of the ethylene glycol (EO) side segments, thereby the side chain length, specifies the properties of POEGMA homopolymers. Brush-like POEGMAs with short-length (of less than ten EO) OEGMA side chains demonstrate thermal responsiveness, but long-length side chains contribute to elevated solubility and enhanced shielding features toward proteins, such as linear poly (ethylene oxide)/poly(ethylene glycol) (PEO/PEG) [33]. 

Controlled radical polymerization (CRP) approaches are among the most facile and widespread synthetic methodologies for the preparation of copolymers as they involve an abundance of monomers to be utilized. Atom–transfer radical polymerization (ATRP), reversible addition–fragmentation chain transfer (RAFT) radical polymerization, and nitroxide-mediated radical polymerization (NMP) have been referred to as efficient radical polymerization techniques [34,35]. In this work, the reversible addition-fragmentation chain transfer (RAFT) polymerization was the synthetic methodology of choice since the less stringent experimental requirements, high structural fidelity of the final product, tolerance to different pendant groups of a vinyl monomer, and maintenance of post-polymerization functionality are some of the highlighted features the RAFT polymerization provides to random copolymer synthesis [11,34].

In the following, we focus on the synthesis and self-assembly behavior of poly(2-(dimethylamino)ethyl methacrylate-co-(oligo ethylene glycol)methacrylate), P(DMAEMA-co-OEGMA), double hydrophilic random copolymers and their subsequent partial post-polymerization functionalization by quaternization. The precursor copolymers were synthesized utilizing a one-step RAFT polymerization procedure, preceded by the chemical modification of DMAEMA tertiary amine groups with alkyl chains of different chain lengths (methyl iodide, 1-iodohexane, 1-iodododecane) hydrophilic/hydrophobic Q_1/6/12_(P(DMAEMA-co-OEGMA) polyelectrolytes. DMAEMA segments ascribed the dual thermo- and pH-responsiveness to the random copolymers whilst following the chemical modification process; they were transformed partially or fully into hydrophilic/hydrophobic segments with permanent cationic charges along the polymer chain of the final copolymer. The P(DMAEMA-co-OEGMA) and Q_1/6/12_(P(DMAEMA-co-OEGMA) random copolymers were characterized in terms of their molecular and physicochemical characteristics, and aggregation properties in aqueous solutions upon heating and at varying pH and ionic strength aiming at studying the copolymer responsiveness to different stimuli.

## 2. Materials and Methods

The monomers 2-(dimethylamino)ethyl methacrylate (DMAEMA, 98%) and (oligo ethylene glycol)methacrylate (OEGMA) (average Mn = 950 g∙mol^−1^, 19 ethylene oxide units) were purchased from Sigma Aldrich, Greece. Both DMAEMA and OEGMA were purified using a column filled with inhibitor removers before polymerization. 2,2′-Azobis (isobutyronitrile) (AIBN), the radical initiator utilized, was purified by recrystallization from methanol. 4-Cyano-4-(dodecylsulfanylthiocarbonyl)pentanoic acid (CDP) as the CTA, methyl iodide (CH_3_I) 1-iodohexane (C_6_H_13_I, ≥98%), 1-iodododecane (C_12_H_25_I, 98%), 1,4-dioxane (≥99.8% pure), and tetrahydrofuran (THF, ≥99.9% pure) were obtained from Sigma Aldrich, Greece and used as received, except 1-4-dioxane, which was first dried over molecular sieves. Deuterated chloroform (CDCl_3_) was used as the solvent for the ^1^H-NMR experiments and was also obtained from Sigma Aldrich, Greece. Dialysis tubing membranes (MEMBRA-CEL^®^) from regenerated cellulose of MWCO 3500 and a diameter of 22 mm were purchased from SERVA, Heidelberg, Germany.

### 2.1. Synthesis of P(DMAEMA-co-OEGMA) Random Copolymers

A one-step reversible addition-fragmentation chain transfer polymerization (RAFT) was applied for the synthesis of poly(2-(dimethylamino)ethyl methacrylate-co-(oligo ethylene glycol)methacrylate) random copolymers, varying at the stoichiometric compositions of each segment. Firstly, DMAEMA and OEGMA monomers were purified by passing through a column filled with monomethyl ether hydroquinone (MEHQ) and butylated hydroxytoluene (BHT) inhibitor removers. The step of OEGMA dissolution in 1,4-dioxane was followed before the purification due to the solid state of the monomer. 4-Cyano-4-(dodecylsulfanylthiocarbonyl)pentanoic acid (CDP) and 2,2′-Azobis (isobutyronitrile) (AIBN) were utilized at 10:1 ratio. For the synthesis of P(DMAEMA-co-OEGMA)_1 copolymer, in a round bottom flask (25 mL) purified DMAEMA (0.8 g, 5.09 mmol), purified OEGMA (1.2 g, 1.26 mmol), CDP (0.04 g, 0.1 mmol), AIBN (0.0016 g, 0.01 mmol) and 3.78 mL 1,4-dioxane (20 wt.% monomer solution) were mixed and dissolved under magnetic stirring. The flask was sealed with a rubber septum, and the mixed solution was degassed by nitrogen gas flow for 20 min and afterward immersed in an oil bath at 70 °C for 24 h under stirring. The polymerization was completed when the flask was placed at −20 °C for 30 min, and finally, the reaction product was exposed to the air. Subsequently, the product was purified from unreacted monomers and other impurities through dialysis against deionized H_2_O for 3 days utilizing dialysis tubing membranes of 3.5 kDa MW. The pure copolymer was isolated after the evaporation of water using a rotor evaporator and dried in a vacuum oven for 48 h at 25 °C. The same methodology was followed for the synthesis of P(DMAEMA-co-OEGMA)_2 copolymers. 

### 2.2. Chemical Modification of P(DMAEMA-co-OEGMA) Random Copolymers

The chemical modification of the random copolymers was accomplished through the quaternization of the tertiary amine group of the DMAEMA segments to quaternary ammonium salt. As quaternization agents, methyl iodide (CH_3_I), 1-iodohexane (C_6_H_13_I), and 1-iodododecane (C_12_H_25_I) were utilized to introduce hydrophilicity/hydrophobicity to the polymeric chain. These agents will be referred to with the prefixes Q_1_, Q_6_, and Q_12,_ respectively, where the subscript presents the number of carbon atoms onto the alkyl side chain of the quaternized DMAEMA segment. The quaternization reaction was achieved when 0.2 g of P(DMAEMA-co-OEGMA) copolymer was dissolved in 10 mL tetrahydrofuran (2% *w*/*v*) in a 25 mL round bottom flask under magnetic stirring, and the quaternization agents were added in different molar ratios. For the chemical modification of P(DMAEMA-co-OEGMA)_1 copolymer 3.69 mmol Q_6_ and Q_12_ for 50% and 1.11 mmol Q_1_ for 100% of stoichiometric quaternization degree, respectively, were added. The methyl iodide agent was added at an excess of 50%. The bottom flask was covered with silver foil because the reactive agents are sensitive to intense light, and the reaction took place at ambient temperature for 24 h for Q_1_ and Q_6_ and 48 h for Q_12_, respectively. The modified copolymers were isolated using a rotary evaporator and dried under a vacuum oven for 48 h. Likewise, Q(P(DMAEMA-co-OEGMA)) modified derivatives (with Q_1_ and Q_6_) were synthesized. 

### 2.3. Self-Assembly of P(DMAEMA-co-OEGMA) Random Copolymers

P(DMAEMA-co-OEGMA) copolymers and their modified derivatives were dissolved in distilled water (polymer concentration 1 × 10^−3^ g/mL), and they were studied for their self-assembly behavior using light scattering techniques after being left overnight at ambient temperature. P(DMAEMA-co-OEGMA) and Q_1/6/12_(P(DMAEMA-co-OEGMA)) solutions were then prepared at three different pHs, i.e., 3, 7, and 10 by addition of an appropriate volume of HCl 0.1 M and NaCl 0.1 M. For the modifications of P(DMAEMA-co-OEGMA)_1 copolymer to the desired pH values, two stock solutions of 1 × 10^−3^ g/mL in distilled water were prepared. Consequently, 100 μL of HCl 0.1 M and 60 μL of NaOH 0.1 M were buffered to each one adjusting the pH value to pH = 3 and pH = 10, respectively. The same methodology was followed for the pH adjustment of each solution, and they were measured after overnight equilibration.

### 2.4. Ionic Strength Studies

The responsive behavior to ionic strength changes was examined through consecutive titration with NaCl 1M. More specifically, nine salt additions led to increasingly different salt concentrations in the polymer solutions. The effect of ionic strength was examined through the scattering intensity and hydrodynamic radius changes using the DLS technique (the measurements were obtained at 90° degrees measuring angle).

### 2.5. Characterization Techniques

#### 2.5.1. Size Exclusive Chromatography (SEC)

The copolymer apparent molecular weight (M_w_) and polydispersity index (M_w_/M_n_) were determined utilizing a Waters chromatography instrument from Waters Technologies Corporation. A Waters 1515 isocratic pump, three μ-Styragel mixed pore separation columns (10^2^ to 10^6^ Å), and a Waters 2414 refractive index detector (equilibrated at 40 °C) were integrated into the instrument. The collected data were analyzed through Breeze software. The eluent was tetrahydrofuran, containing 5% *v*/*v* triethylamine, at a flow rate of 1 mL/min and 30 °C. For the SEC analysis, 2–4 mg/mL of each P(DMAEMA-co-OEGMA) copolymer was dissolved in tetrahydrofuran.

#### 2.5.2. Proton Nuclear Magnetic Resonance Spectroscopy (^1^H-NMR)

The chemical structure, the mass composition (% wt.) of P(DMAEMA-co-OEGMA) copolymers, and the quaternization degree of the modified derivatives were verified through ^1^H-NMR spectroscopy. The ^1^H-NMR experiments were conducted on a Varian 300 MHz spectrometer (Palo Alto, CA, USA), and tetramethylsilane (TMS) was used as the internal standard in deuterated chloroform (CDCl_3_) and deuterated oxide (D_2_O). The samples were prepared by the dissolution of 0.0100 g of the dry copolymer in 0.700 mL of solvent.

#### 2.5.3. Light-Scattering 

The light-scattering studies were conducted to determine the size, size polydispersity, surface charge, and morphology of the formed copolymer aggregates when exposed to different pH and temperatures. Dynamic light scattering (DLS) experiments were carried out on an ALV/CGS-3 compact goniometer system (ALVGmbH, Hessen, Germany) equipped with an ALV 5000/EPP multi-τ digital correlator with 288 channels, an ALV/LSE-5003 light-scattering electronics unit for a stepper motor drive and a JDS Uniphase 22 mW He-Ne laser (λ = 632.8 nm), as the light source. The calibration was performed using toluene as the standard solvent. The copolymer solutions were loaded into cylindrical optical glass cuvettes after being filtered through 0.45 µm hydrophilic PVDF filters (Millipore, Billerica, MA, USA) for the removal of dust particles. The measurements were conducted at an angular range of 45 to 135 degrees and a temperature range of 25 to 55 °C. For each angle, five measurements of 30 s were executed. The obtained autocorrelation functions were analyzed via the cumulants method and CONTIN algorithm. Moreover, the determination of the effective surface charge of the aggregates in solutions was performed on a ZetaSizer Nano series 11 Nano-ZS (Malvern Instruments Ltd., Malvern, UK) equipped with a He-Ne laser at a wavelength of 633 nm and a fixed backscattering angle of 173°. The Henry correction of the Smoluchowski equation was used for the analysis of zeta-potential values after equilibration at 25 °C. Each measurement was an average of 50 scans. 

#### 2.5.4. Fluorescence Spectroscopy (FS)

Fluorescence spectroscopy measurements were conducted to evaluate the micropolarity of the copolymers in water at different pHs. The fluorescence spectra were recorded on a Fluorolog-3 JobinYvon-Spex spectrofluorometer (model GL3–21, Kyoto, Japan) in the range 355 to 640 nm. Pyrene was utilized as the fluorescent probe due to its sensitivity to the polarity of the environment within the aggregates formed in each case. The excitation wavelength for the measurements was 335 nm. Copolymer solutions at concentrations of 10^−3^ g/mL were prepared via the direct dissolution of a specific quantity of the copolymer into distilled water. Typically, 1 μL pyrene stock solution (1 mM) in acetone was added for each 1 mL of the polymer solution and allowed to equilibrate. 

## 3. Results

### 3.1. Synthesis and Molecular Characterization of P(DMAEMA-co-OEGMA) Copolymers and Their Quaternized Derivatives

**Scheme 1:**
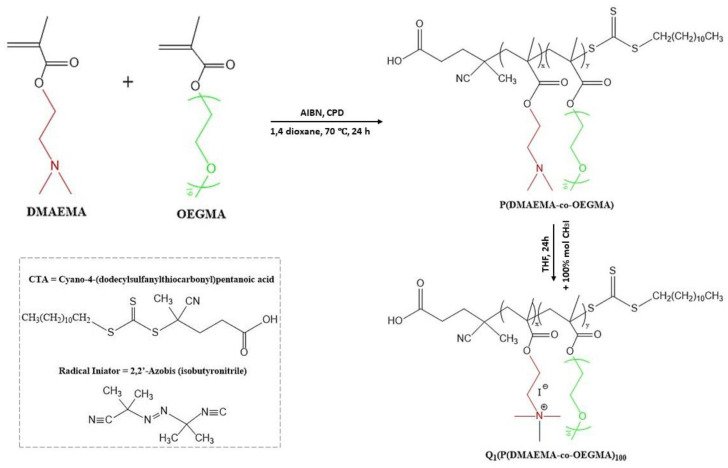
Synthetic route of P(DMAEMA-co-OEGMA) random copolymer via one-step RAFT polymerization followed by the hydrophilic chemical modification with methyl iodide (CH_3_I).

**Figure 1:**
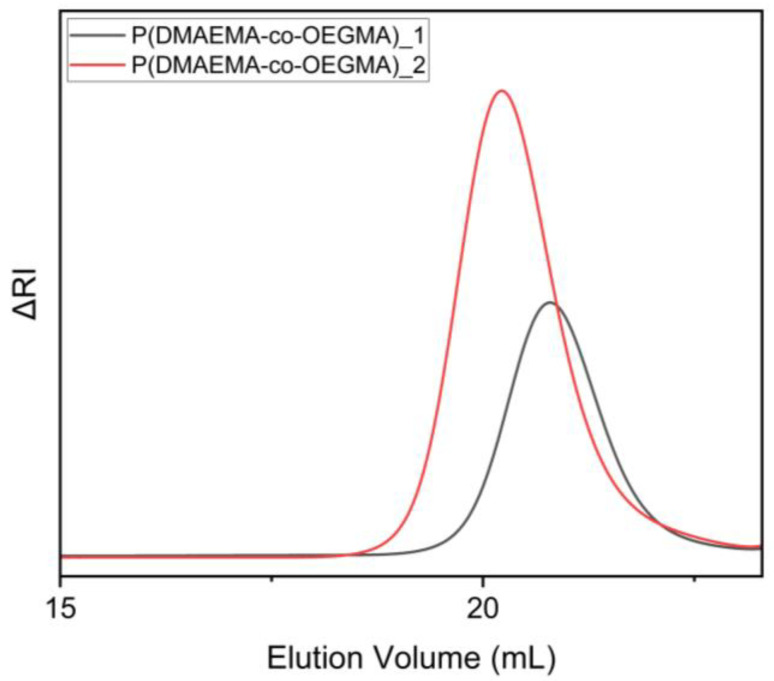
Size exclusion chromatography traces of P(DMAEMA-co-OEGMA)_1 and P(DMAEMA-co-OEGMA)_2 copolymers in tetrahydrofuran (THF) containing 5% Et_3_N.

Two poly(2-(dimethylamino) ethyl methacrylate-co-(oligo ethylene glycol) methacrylate) double hydrophilic random copolymers were synthesized via a one-step RAFT polymerization procedure (Scheme 1). Oligo (ethylene glycol) methacrylate with M_n_ = 950 g∙mol^−1^ (19 ethylene oxide units) was the oligomer of choice due to the enhanced colloidal stability and shielding properties that it can confer to the final product. 4-Cyano-4-(dodecylsulfanylthiocarbonyl)pentanoic acid (CDP) was selected as the CTA since it is compatible with methacrylate-based monomers [36]. The copolymer's apparent molecular weight and molecular weight distributions were determined through size-exclusion chromatography (SEC). The chromatogram (Figure 1) reveals the efficient purification of P(DMAEMA-co-OEGMA) copolymers from unreacted OEGMA monomers since they were taken after dialysis against deionized H_2_O. The peaks for both copolymers are monomodal and relatively narrow, with the minimum amount of tails. The extracted polydispersities are within the range usually reported for random copolymers obtained by RAFT polymerization processes [36]. The difference beyond the elution time for each copolymer is ascribed to the size of polymer molecules and, thus, to the molar mass. The apparent molecular weights and the dispersity indexes of the two copolymers are given in Table 1.

The double-hydrophilic P(DMAEMA-co-OEGMA) random copolymers were chemically modified, intended to be converted into strong polyelectrolytes with enhanced hydrophobicity and permanent effective surface charges. By employing this simple and efficient post-polymerization modification, the tertiary amine group of the DMAEMA segment was converted to positively charged quaternary ammonium salt. The careful selection of the functional alkyl halides (X-(CH_2_)q-R) with different alkyl chain lengths, namely the methyl iodide, 1-iodohexane, and 1-iodododecane in this study, a series of copolymers with different hydrophobic/hydrophilic balance and molecular weight/composition was produced. It is anticipated that the alkylated quaternary group will offer enhanced hydrophobicity to the copolymer and, depending on the aiming quaternization degree, partially or completely obscure the pH and thermal-induced behavior typical for PDMAEMA homopolymer. In addition, the random distribution of cationic charges along the polymer chains, in principle, enables them to be utilized in protein and gene delivery systems through electrostatic complexation with the protein/nucleic acid cargo. It should be noted that P(DMAEMA-co-OEGMA)_2 copolymer and its quaternized derivatives have been successfully studied for the complexation with ovalbumin in our previous study [37]. Cationic groups also provide antimicrobial activity [38] to the macromolecules enhancing the potential application of the present copolymers. P(DMAEMA-co-OEGMA) were modified at 50% and 100% molar ratios (based on stoichiometry) using 1-iodohexane (C_6_H_13_I) and methyl iodide (CH_3_I), respectively. 1-iodododecane (C_12_H_25_I) was utilized as a quaternizing agent only for the partial modification of P(DMAEMA-co-OEGMA)_1 copolymer. The P(DMAEMA-co-OEGMA)_2 is comprised of a higher percentage of the DMAEMA segment; hence the attachment of twelve carbon atoms to the alkyl chain will mask its stimuli-responsive behavior and make it insoluble in water. Eventually, the distinguishing factors of each modified copolymer are the number of attached alkyl chains and, thus, the quaternization degree, further affecting the molecular characteristics and solution properties of the copolymers.

**Figure 2:**
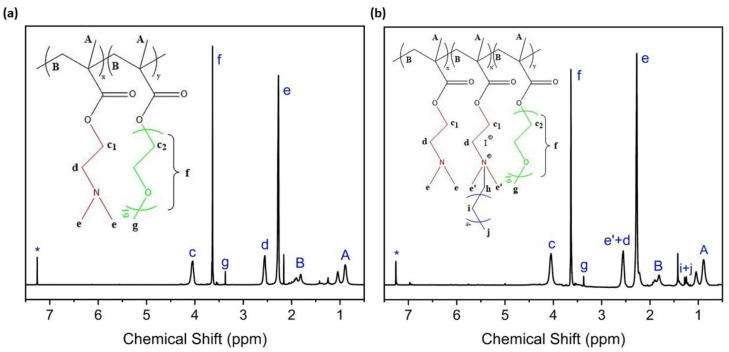
^1^H-NMR spectra for (**a**) P(DMAEMA-co-OEGMA) random copolymer and (**b**) Q_6_(P(DMAEMA-co-OEGMA)_2)_39_ in deuterated chloroform (CDCl_3_) (which is denoted with (*) in the spectra).

^1^H-NMR spectroscopy was applied to verify the chemical structure and composition of the chemically modified random copolymers as well as their precursors. Figure 2 displays the ^1^H-NMR spectrum for (a) P(DMAEMA-co-OEGMA)_2 double hydrophilic random copolymers and its modified derivative (b) Q_6_(P(DMAEMA-co-OEGMA)_2). The chemical composition of each segment of the P(DMAEMA-co-OEGMA) copolymer was determined through the estimation of the integrals of the characteristic peaks. Nevertheless, because of the possible overlapping between the spectral peaks of the OEGMA ethylene glycol side chain (peak f at 3.63 ppm, -(CH_2_CH_2_O)_9_-)) and 1,4-dioxane (peak at 3.69 ppm), which was the solvent for the RAFT polymerization, the -CH_3_ protons at 3.36 ppm (peak g, -(CH_2_CH_2_O)_9_CH_3_-)) and the -N(CH_3_)_2_- protons at 2.17 ppm of DMAEMA segment were utilized for the calculation of chemical composition. The obtained mass compositions were close to the stoichiometric values (Table 1). The characteristic spectral peaks of Q_6_DMAEMA, DMAEMA, and OEGMA segments are observed at 2.55 ppm (peak e’, 8H, -N^+^((CH_3_)_2_(CH_2_))-), 2.28 ppm (peak e, 6H, -N(CH_3_)_2_-) and 3.63 ppm (peak f, 4H, -(CH_2_CH_2_O)_9_-) and belong to the -CH_3_ protons of quaternary amino groups of the Q_6_PDMAEMA segment, the CH_3_ protons of the tertiary amine group of DMAEMA segment and the -CH_2_ protons of the ethylene glycol side chain of OEGMA monomer, respectively. Yet, comparing the ^1^H-NMR spectra of the polyelectrolyte with its double hydrophilic precursor in the same spectral region in which the -OCH_2_CH_2_N protons at 2.55 ppm (peak d) (Figure 2a) are presented, the protons of quaternary amino groups are anticipated to be detected [28]. Hence, the overlapping between e and d signals renders the calculations of chemical compositions inaccurate. The ^1^H-NMR spectroscopy in this case was utilized for the qualitative detection of changes as a consequence of the modification process. Moreover, the spectra of polyelectrolytes with methyl iodide as a quaternizing agent were obtained using deuterium oxide (D_2_O) as the solvent since, in deuterated chloride, they were insoluble. The quaternization degree for each modification was obtained by rationing the integrals of the spectral peaks that correspond to the dimethylamino protons of the DMAEMA segments and the cationic quaternary amino groups of QPDMAEMA residues.

### 3.2. Internal Micropolarity Studies of Polyelectrolytes Using Pyrene Assay

Fluorescence spectroscopy measurements were conducted in order to investigate the internal micropolarity of nano aggregates in aqueous solutions utilizing pyrene as the hydrophobic probe and distilled water as the solvent. Pyrene has low solubility in water, and because of its hydrophobic character, it can be easily incorporated into hydrophobic nanodomains of polymer aggregates and provide qualitative information about their polarity. The ratio between the first and the third vibronic peak (I_1_/I_3_) indicates the microenvironment polarity; therefore, the ratio decreases with the reduced polarity of the media [39]. The nanostructure’s internal polarity was estimated by evaluating the ratio I_1_/I_3_ at 25 °C and pH = 7 for the double hydrophilic copolymers and pH = 3, 7, and 10 for the quaternized derivatives to verify if the modification process affected their conformation at different pH environments. 

According to the results from fluorescence spectroscopy measurements, both P(DMAEMA-co-OEGMA)_1 and P(DMAEMA-co-OEGMA)_2 demonstrated elevated I_1_/I_3_ (ca. 1.63 and 1.59 respectively), but lower than the value for pyrene in water (ca. 1.8–1.9). The moderate difference in polarity of P(DMAEMA-co-OEMA)_2 compared to the P(DMAEMA-co-OEGMA)_1 copolymer is potentially correlated with the higher content in DMAEMA segments (76% wt. and 42% wt., respectively, Table 1) that tends to self-fold in the inner domains. Hence, the conformation of the copolymers is rather loose, judging from the hydrophilic internal micropolarity. Moving on to the microenvironment of modified derivatives, they showed a comparable tendency in the internal polarity shifting from neutral to acidic and basic pH values (Table 2). Concerning the partially modified derivatives, when the solution is acidic, the dimethylamino groups of unmodified DMAEMA segments are fully protonated, increasing the system’s hydrophilicity and thus the I_1_/I_3_ values. Deprotonation of amino groups, on the other hand, results in an increase in copolymer hydrophobicity at pH = 10, which is associated with a decrease in I_1_/I_3_ ratio as a result of pyrene encapsulation into more hydrophobic domains of existing copolymer aggregates. However, the polyelectrolytes are estimated to self-assemble intermolecularly into hydrophilic nanostructures of rather a loose arrangement, where aqueous areas are located in the inner domain consisting of hydrophobic chains, judging from the I_1_/I_3_ values. It is noteworthy that Q_12_(P(DMAEMA-co-OEGMA)_1)_50_ organized into well-arranged nanoassemblies since the pyrene was enclosed in an environment of reduced polarity (Figure S1). Ultimately, the alkyl chains pendant to the main chains controls the way polymer chains self-assemble in aqueous solutions. Nonetheless, light scattering measurements were conducted to shed light on the self-assembly behavior upon exposure to different stimuli and to verify these assumptions (the results are presented in the following section). 

### 3.3. Self-Assembly Studies as a Function of Different Stimuli by Light Scattering Techniques

**Figure 3:**
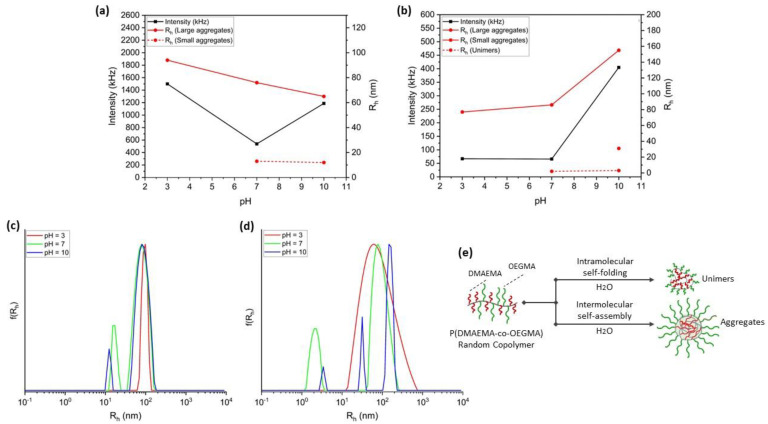
pH-responsive behavior of (**a**) P(DMAEMA-co-OEGMA)_1) and (**b**) P(DMAEMA-co-OEGMA)_2 double hydrophilic copolymers observed through scattering intensity and hydrodynamic radius obtained from DLS measurements. Distributions of hydrodynamic radius obtained from CONTIN analysis (at θ = 90° and pH 3, 7, and 10) for (**c**) P(DMAEMA-co-OEGMA)_1 and (**d**) P(DMAEMA-co-OEGMA)_2 copolymers. (**e**) Graphical illustration of self-folding/self-assembly of P(DMAEMA-co-OEGMA) random copolymer dissolved in distilled H_2_O.

Random copolymers are typically self-folded intramolecularly when added to aqueous solutions, resulting in unimolecular nanoaggregates with micellar-like structures. The self-assembly behavior of random copolymers is influenced by the hydrophobic/hydrophilic ratio and chain length. Unimers, i.e., single-chain structures with hydrophobic inner nanodomains, arise due to intramolecular self-folding, specifically when the content of the hydrophobic segments is low. Instead, as the hydrophobic content rises, multichain aggregates are created as a result of intermolecular self-assembly (Figure 3e) [40]. To elucidate the copolymer self-folding/self-assembly behavior in aqueous solutions at room temperature, light scattering studies were conducted on aqueous copolymer solutions. The measurement data for copolymer solutions with acidic, neutral, and basic pH are listed in Table 3 and Table 4 below. The size polydispersity index (PDI) was obtained from the cumulants method, whilst CONTIN analysis was used to determine the hydrodynamic radius (R_h_) distribution. The scattering intensity is a susceptible probe depending on the scattering angle and molar mass; thus, sudden shifts in its magnitude are sufficient evidence of transitions that have an impact on the copolymer self-assembly [41]. 

#### 3.3.1. pH-Responsiveness of Double Hydrophilic P(DMAEMA-co-OEGMA) Precursors

The PDMAEMA homopolymer performs as a weak polyelectrolyte with a positive charge and strong water solubility when the pH is neutral. The tertiary amine groups are fully protonated at pH < 6, yielding strongly charged polymer nanodomains with increased solubility in water. On the other hand, whenever the pH is basic (pH > 8), dimethylamino groups are almost entirely deprotonated, producing nanoaggregates in the aqueous solutions [42]. Acknowledging that polymers comprised of DMAEMA segments are anticipated to present conformational and aggregation state changes when exposed to different solution pH values, the P(DMAEMA-co-OEGMA) copolymers have comprehensively been studied using light scattering techniques.

It is revealed that both double hydrophilic copolymers exhibited pH responsiveness but with the most outstanding one for P(DMAEMA-co-OEGMA)_2. At neutral aqueous solutions, the copolymers demonstrated bimodal size distributions indicating the coexistence of two kinds of populations (Figure 3c,d). Comparing their hydrodynamic radius and contrasting it with the recorded scattering intensity, one could deduce that the copolymer of lower DMAEMA content creates multichain aggregates of different sizes with high apparent molar mass (I = 538 kHz). On the other hand, the solution of P(DMAEMA-co-OEGMA)_2 copolymer contains aggregates that scatter less and have lower mass (I = 66 kHz) but with higher size (R_h_ = 86 nm) and heterogeneity (PDI = 0.59). It should be noted that in the case of P(DMAEMA-co-OEGMA)_1, the smaller in size formed nanoaggregates (R_h_ = 13 nm) may have arisen from the intermolecular self-assembly of a small number of copolymer chains (Figure 3a), whilst unimers (R_h_ = 2 nm) due to the intramolecular self-folding are formed by the P(DMAEMA-co-OEGMA)_2 copolymer (Figure 3b). When the pH is changed from neutral to acidic, the hydrodynamic radius of P(DMAEMA-co-OEGMA)_1 is marginally increased, and so is the intensity, indicating the formation of most probably hydrophilic aggregates with one type of species present in the solution, as a result of the protonated (cationic) group of DMAEMA segment. Instead, in an acidic environment P(DMAEMA-co-OEGMA)_2 chains were further aggregated to larger assemblies (R_h_ = 77 nm). Yet, the presence of two monomers with high affinity to water at neutral pH does not further affect the hydrophilicity of the random copolymer, and so protonation of the dimethyl amino groups at acidic pH provokes disaggregation phenomena. Nevertheless, the complex behavior of random copolymers, as well as the ratio of hydrophilic/hydrophobic degrees, are of paramount importance parameters that influence the products’ responsiveness. One could assume that the increase of both aggregate size and mass at pH ca. 3 for P(DMAEMA-co-OEGMA)_1 could be attributed to hydrophilic interactions, which, however, are rather well-defined as the low-PDI value (ca. 0.12) indicates. Regarding the responsiveness to the basic environment, both copolymers exhibited similar behavior. The complete deprotonation of the amino groups leads to the generation of large aggregates, which are expected to be loose in structure. The striking increase in intensities justifies the presence of nanostructures higher in size and mass compared to those formed at pH 7. In addition, the structural complexity both of copolymers is ascribed to the high PDI values (Table 2). Notably, P(DMAEMA-co-OEGMA)_2 at basic environment generated intermolecularly smaller in size aggregates (R_h_ = 31 nm) and unimers (R_h_ = 3 nm).

Zeta-potential values were determined through ELS measurements and validated the effective surface charge of the nanoassemblies. The surface charge at neutral and acidic environments was positive for both double hydrophilic copolymers owning to partial and full protonation of the dimethylamino groups at the corresponding pH values. On the other hand, buffering to pH ca. 10 resulted in negative zeta-potential values (ζ_p_ = −36 ± 5.6, ζ_p_ = −23 ± 7.3). An explanation of the negative surface charges at basic pH relates to the absorption of hydroxyl ions on the particle surface. Additionally, the COOH group of CTA located at the chain terminal converted into an anionic carboxylate group attached to the surface of the aggregate when the random copolymers self-assemble at basic conditions [43].

**Table 3:** Physicochemical characteristics of P(DMAEMA-co-OEGMA)_1 and P(DMAEMA-co-OEGMA)_2 double-hydrophilic copolymers.

Sample	pH	Intensity ^a^ (kHz)	R_h_ ^a^ (nm)	PDI ^a^	Zeta-Potential ^b^ (mV)
P(DMAEMA-co-OEGMA)_1	3	1500	94	0.12	+38 ± 5.9
	7	538	13/76	0.24	+90 ± 8.8
	10	1187	12/83	0.33	−36 ± 5.6
P(DMAEMA-co-OEGMA)_2	3	67	77	0.46	+32 ± 8.7
	7	66	2/86	0.59	+10 ± 3.8
	10	405	3/31/155	0.51	−23 ± 7.3

^a^ Determined by DLS at measuring angle 90° and temperature 25 °C. The Intensity values are determined with 1–2% error and the R_h_ values with 5%; ^b^ Determined by ELS. Note: The different R_h_ values correspond to different populations detected in the aqueous solution. The smaller values of P(DMAEMA-co-OEGMA)_1 and P(DMAEMA-co-OEGMA)_2 solutions signify the presence of smaller in size aggregates formed by single copolymer chains or aggregates of a small number of copolymer chains. The population with a larger R_h_ in both solutions is assigned to large multichain aggregates.

#### 3.3.2. pH-Responsiveness of Q(P(DMAEMA-co-OEGMA)) Polyelectrolytes Modified with Alkyl Chains

Taking into account that the quaternization reaction is a chemical modification that involves alkyl chains anchored to the amine groups of DMAEMA segments, yielding both enhanced hydrophobic/hydrophilic profile and effective positive charge, it is anticipated that different pHs will influence the nanoassemblies and their stability in diverse ways. The polyelectrolytes’ conformation in water arises from the chain folding through the self-assembly of hydrophobic alkyl pendant groups and hydrophobic backbones into the inner domains, whilst the ethylene glycol side chains envelope and stabilize the hydrophobic inner domain. The length of the alkyl chains and the balance between hydrophilic and hydrophobic components prompts the formation of random copolymer aggregates with several physicochemical features in different aqueous environments. Nevertheless, due to the random distribution of the hydrophobic side chains along the copolymer chain, it is unclear whether a well-clustered hydrophobic core is created or if numerous hydrophobic nanodomains coexist within the aggregate’s volume. 

**Figure 4:**
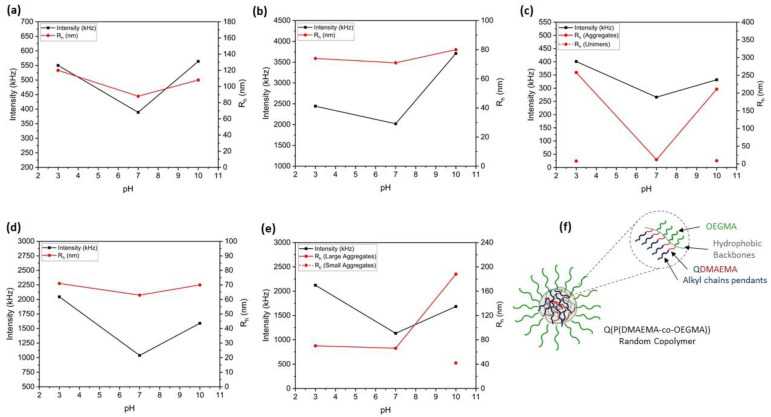
pH-responsive behavior of (**a**) Q_1_(P(DMAEMA-co-OEGMA)_1))_100_, (**b**) Q_6_(P(DMAEMA-co-OEGMA)_1))_39_, (**c**) Q_12_(P(DMAEMA-co-OEGMA)_1))_50_, (**d**) Q_1_(P(DMAEMA-co-OEGMA)_2))_100_, (**e**) Q_6_(P(DMAEMA-co-OEGMA)_2))_50_ chemically modified random copolymers as observed by scattering intensity and hydrodynamic radius changes from DLS measurements. (**f**) Graphical illustration of Q(P(DMAEMA-co-OEGMA) random copolymer self-assembly into a micelle-like structure with an inner hydrophobic domain (comprised of methacrylate-type backbones and alkyl chains attached to DMAEMA segment) shielded by hydrophilic OEGMA segment.

The majority of quaternized derivatives revealed similar features when inserted into neutral aqueous solutions as far as the aggregate mass is concerned (Table 4). At pH 7, the modified copolymers intermolecularly self-assembled into aggregates giving rise to significantly increased scattering intensity, indicating the association of a larger number of copolymer chains into more compact structures, compared to their precursors, with rather smaller dimensions as a result of amplified hydrophobic interactions occurring due to the QDMAEMA segments. Yet, Q_1_(P(DMAEMA-co-OEGMA)_1)_100_ copolymer formed less dense nanostructures (I = 389 kHz), but higher in hydrodynamic radius (R_h_ = 88 nm) (Figure 4a). One could infer that this conformation is correlated with the short alkyl chain (one methyl is attached to nitrogen), whereupon the ethylene oxide units of OEGMA segments cover the hydrophobic domain minimizing the aqueous interface (Figure 4f). Eventually, the absence of two types of aggregate populations in all solutions (at pH 7), accompanied by relatively low PDI values and monomodal size distributions (Figure S2a), evidence that hydrophobicity provoked the participation of all copolymer chains in the creation of the aggregates.

**Table 4:** Physicochemical features of chemically modified derivatives of Q(P(DMAEMA-co-OEGMA)_1) and Q(P(DMAEMA-co-OEGMA)_2) random copolymers.

Sample	pH	Intensity ^a^ (kHz)	R_h_ ^a^ (nm)	PDI ^a^	Zeta-Potential ^b^ (mV)
Q_1_(P(DMAEMA-co-OEGMA)_1)_100_	3	550	120	0.34	+50 ± 2.5
	7	389	88	0.27	+51 ± 8.1
	10	564	108	0.26	+42 ± 1.2
Q_6_(P(DMAEMA-co-OEGMA)_1)_39_	3	2447	74	0.14	+63 ± 8.5
	7	2019	71	0.16	+66 ± 7.6
	10	3710	80	0.19	+34 ± 5.5
Q_12_(P(DMAEMA-co-OEGMA)_1)_50_	3	401	8/258	0.47	+20 ± 8.6
	7	266	12	0.41	+21 ± 10.8
	10	332	9/211	0.45	+14 ± 7.3
Q_1_(P(DMAEMA-co-OEGMA)_2)_100_	3	2045	71	0.17	+21 ± 6.0
	7	1037	63	0.18	+22 ± 6.3
	10	1590	70	0.20	+5 ± 1.34
Q_6_(P(DMAEMA-co-OEGMA)_2)_50_	3	2123	70	0.12	+17 ± 2.8
	7	1132	66	0.23	+21 ± 2.0
	10	1684	42/188	0.30	+6 ± 1.61

^a^ Determined by DLS at angle of 90° and temperature of 25 °C. The Intensity values are determined with 1–2% error and the R_h_ with 5%; ^b^ Determined by ELS. Note: Copolymers concentration is 10^−3^ g∙mol^−1^. The different R_h_ values correspond to differences in size aggregates detected in the aqueous solutions of Q_6_(P(DMAEMA-co-OEGMA)_1)_50_ and Q_12_(P(DMAEMA-co-OEGMA)_1)_50_ copolymers.

When the pH is changed to acidic and basic, respectively, Q_1_(P(DMAEMA-co-OEGMA_1)_100_ and Q_1_(P(DMAEMA-co-OEGMA_2)_100_ demonstrate a similar tendency on how mass and size fluctuate, since both parameters change to higher values (Table 4), signifying the establishment of potentially dense aggregates. The complete modification of dimethylamino groups to ammonium salts prevents their response to pH. Nonetheless, the attachment of one methyl group on the DMAEMA segment renders the nanoaggregates rather hydrophilic but well-clustered, justified by their low PDI values. Likewise, the adjustment of pH at ca. 3 and ca. 10 for partially modified derivatives with 1-iodohexane as the quaternized agent (Figure 4b,e) provokes the formation of more compact aggregates containing a larger number of copolymer chains (since the scattering intensity increases) and slightly higher in size as a result of amplified copolymer hydrophobicity. Obviously, the agglomeration at basic media is enhanced by the hydrophobic interactions from the long alkyl chains, but at this pH Q_6_(P(DMAEMA-co-OEGMA_2)_50_ disaggregated concurrently with the self-assembly into different in size multichain aggregates (R_h_ = 48/188 nm) producing rather heterogeneous aggregates (PDI = 0.30). Last but not least, the partial modification of P(DMAEMA-co-OEGMA)_1 precursor with 1-iododecane results in the formation of small aggregates (R_h_ = 11 nm) at neutral pH, owing to the amplified hydrophobicity but with visible disaggregation phenomena when shifting pH to acidic and basic conditions before the remarkable growth of their size (Figure 4c). The bimodal size distributions (Figure S2c) are accompanied by large-size heterogeneity (PDI ≈ 0.4), in which the smaller-size nanostructures potentially belong either to aggregates formed by single copolymer chains (unimers) or aggregates of a small number of copolymer chains.

The zeta-potential values obtained through ELS measurements corroborated the cationic nature of the formed nanoaggregates. The positive values arising from the partial/complete modifications are justified by the persistent cationic charges of quaternized derivatives. Even so, aggregation phenomena affect the surface charge. Apropos, the presence of some ζ_p_ values close to zero in basic conditions for Q(P(DMAEMA-co-OEGMA)_2) ((+5 ± 1.34 mV), (+6 ± 1.61 mV)) are probably ascribed to the fact that the positively charged DMAEMA segments are hidden in the inner domain shielded by the OEGMA segments (Figure 4f) whilst hydroxyl group ions can be absorbed on the aggregate’s surface. It should not be neglected that at pH=10, deprotonation of non-quaternized amino groups of DMAEMA segments occurred, further amplifying the hydrophobic character of the chains and gaining negative charges on the surface, probably leading to charge neutralization. 

Collating the data originating from DLS and ELS measurements, the random placement of hydrophilic/hydrophobic groups, the length of attached alkyl pendant groups, and the quaternization degree strongly determine the way these random polyelectrolytes respond at different pH solutions. Each of the solutions demonstrated substantial conformational changes, especially as far as the aggregate mass and the density are concerned. In most cases, the copolymers are further aggregated upon exposure to different pHs, whilst more ethylene oxide units of OEGMA segments shield the hydrophobic inner domains. Conclusively, the present polyelectrolytes exhibited well-arranged domains justified by the low PDI values and the narrow size distributions, even though the hydrophilic/hydrophobic domains interface of random copolymer aggregates may not be precisely defined, as in the case of spherical block copolymer micelles.

#### 3.3.3. Thermo-Responsiveness of Self-Assembled Random Copolymers

Stimuli-responsive polymers can be manipulated in terms of morphological configurations, solubility, and self-assembly upon a temperature change. The polymers that can undergo phase transition may exhibit lower critical solution temperature (LCST). Heating above a critical point (cloud point, Cp) provokes the solution’s turbidity or even phase separation, whilst cooling below Cp causes the system to present reversible homogeneity [23]. Polymers that present LCST-type solubility in water, such as poly(2-(dimethylamino) ethyl methacrylate) (PDMAEMA), are mainly comprised of hydrophobic and hydrophilic segments. The hydrophobic to the hydrophilic balance of polymer chains and the induced hydrophobicity of hydrophilic parts affect the cloud point temperatures of the polymers. Moreover, the Cp temperatures of such polymers are influenced by other factors, including solvent quality, salt addition, pH, and polymer concentration [44]. 

**Figure 5:**
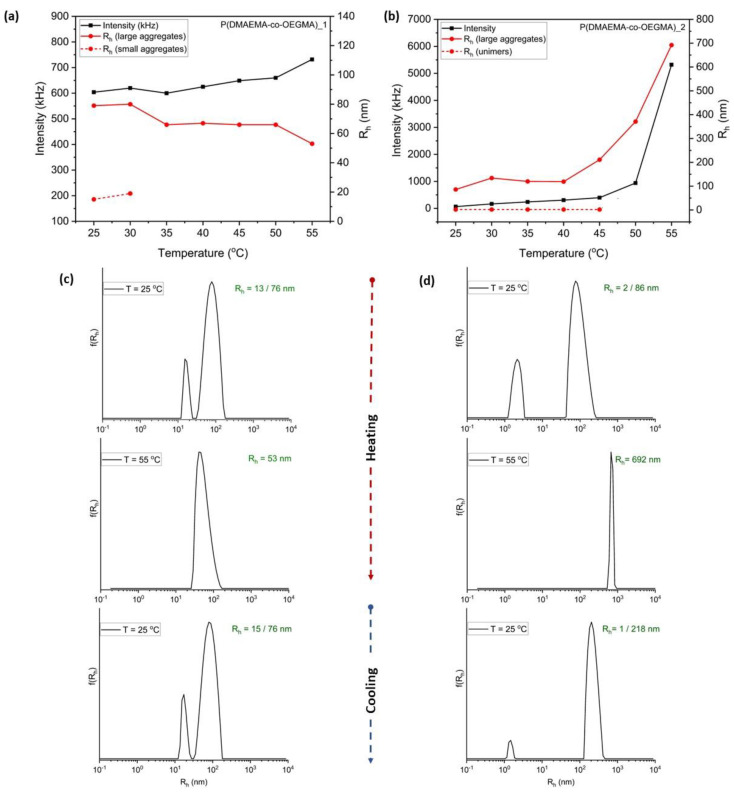
Correlation of scattering intensity and hydrodynamic radius of (**a**) P(DMAEMA-co-OEGMA)_1 and (**b**) P(DMAEMA-co-OEGMA) upon temperature increase (at θ = 90° and pH = 7) and (**c**), (**d**) size distributions from CONTIN analysis at heating–cooling cycle (from 25 °C to 55 °C and reverse) obtained from DLS measurements.

Dynamic light scattering measurements of the copolymer solutions were conducted at different temperatures to document the phase transition, if any. Amongst the double-hydrophilic copolymers, P(DMAEMA-co-OEGMA)_2 exhibited significant changes in mass and size of forming nanostructures, given the fact that it contains a higher DMAEMA mass. Observation of the scattering intensity of both copolymer solutions (Figure 5b) certified that the phase transition to a dehydrated state is accomplished above 30 °C. Even though they follow a similar tendency on how scattering intensity, and thus mass, is changing as a function of temperature, the detected values of P(DMAEMA-co-OEGMA)_2 show a more intense increase. Notably, the above-mentioned random copolymer at 25 °C self-folded intramolecularly into unimers (R_h_ = 2 nm) and self-assembled intermolecular multichain aggregates (R_h_ = 86 nm) with low overall mass (I = 66 kHz). By gradually increasing the temperature, size and mass are exceptionally elevated. Nevertheless, in the range of 30 to 40 °C, a slight size collapse is detected, owing to the system’s tendency for agglomeration that leads to shrinkage of multichain aggregates. Hereupon, the continuous rise of hydrodynamic radius coincides with the recorded increasing scattering intensity upon heating, further indicating the occurring secondary aggregation phenomena. The intermolecular chain aggregation increases the recorded scattering intensity until the cloud point (T = 55 °C in our system), in which amplified hydrophobicity is immerging. Generally, increased temperature weakens hydrogen bonds, partially dehydrates polymer chains and inhibits further solubilization, which promotes polymer aggregation, a phenomenon inferred from the intensified ordering that unfavorably affects the entropy of mixing [45]. The absence of hydrated single copolymer chains above 45 °C should also be noted. These coiled-like chain conformations tend to minimize their contact with the surrounding water molecules by transitioning toward globular-like structures or even interacting with the pre-existing copolymer aggregates to form few micrometers multichain assemblies (R_h_ = 692 nm) in a rather monodisperse nanoassembly (PDI = 0.13). Hence, this assumption is verified by the size distributions from CONTIN analysis (Figure 5d) at T = 55 °C, in which a narrow peak is recorded. On the other hand, P(DMAEMA-co-OEGMA)_1 copolymer seems to be less thermoresponsive within the temperature range from 25 to 55 °C (Figure 5a,c). Above the assumed dehydrated state at 30 °C, the small aggregates are further assembled with the large ones, increasing the overall mass (Table 5) and decreasing the observed hydrodynamic radius. One could deduce that the presence of a higher percentage of oligo ethylene oxide side chains of the OEGMA segments provides enhanced hydrophilicity, yet enough to shield the hydrophobic inner domain and mask, to some extent, the response to temperature increase. In conclusion, it can be stated that both hydrophilic copolymers demonstrated different scales of temperature-responsive behavior, rather reversible, after the heating–cooling cycle (at least for the case of P(DMAEMA-co-OEGMA)_1 copolymer). 

**Figure 6:**
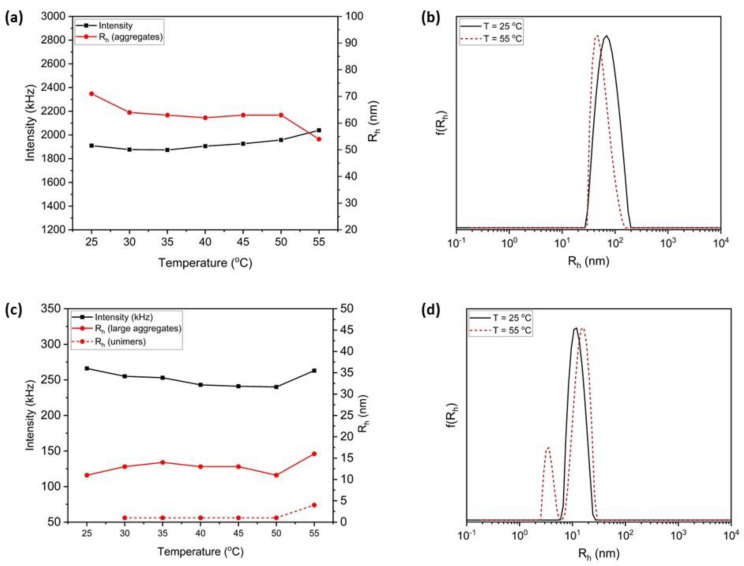
Dependence of scattering intensity and hydrodynamic radius of (**a**) Q_6_(P(DMAEMA-co-OEGMA)_1)_39_ and (**c**) Q_12_(P(DMAEMA-co-OEGMA)_1)_50_ upon temperature (at θ = 90° and pH = 7) and (**b**), (**d**) size distributions from CONTIN analysis at T = 25 °C and T = 55 °C obtained from DLS measurements.

The hydrophilic to the hydrophobic balance of the copolymers, as defined by the copolymer composition, the length of hydrophobic alkyl pendant groups or hydrophilic ethylene glycol chains and the backbone structures (methacrylate-based vs. acrylate-based), can also influence and tune the cloud point and thus the response to temperature [44]. The amplified permanent hydrophobicity induced by the chemical modification with alkyl chains of different carbon–atom numbers masks the effects of temperature on the conformation/aggregation state of the copolymers. In this regard, heating the aqueous solutions will provoke amplified hydrophobic forces among the hydrophobic groups, resulting in an elevation of aggregate mass and a decrease in size [45]. Since the mass composition of the precursor double hydrophilic copolymer was fewer than that of the second copolymer, the temperature-responsive behavior of Q_6_(P(DMAEMA-co-OEGMA)_1)_39_ and Q_12_(P(DMAEMA-co-OEGMA)_1)_50_ copolymers was subsequently scrutinized (Table 6). As far as the scattering intensity of both hydrophobic derivatives is concerned, the phase transition to a more dehydrated state is located above 30 °C. The overall changes throughout the heating process are not significant, however, with a different response at the transition from 50 to 55 °C. Particularly, the scattering intensity of Q_6_(P(DMAEMA-co-OEGMA)_1)_39_ polyelectrolyte followed a similar tendency with the hydrodynamic radius, and one aggregate population was observed at all temperatures. A slight rise in scattering intensity was observed from 50 to 55 °C, further confirming the secondary chain aggregation (Figure 6a). The alkylated quaternary moieties are not interacting with water molecules via hydrogen bonding, inducing aggregation phenomena driven by the hydrophobic interactions, whereupon they are clustered into hydrophobic domains. The moderate decrease in aggregates’ sizes at 55 °C is conceivably ascribed to the weakening of hydrogen bonds between DMAEMA parts and water molecules since the polyelectrolytes are not fully quaternized. Moreover, the low PDI value accompanied by the narrow monomodal size distributions (Figure 6b) confirmed that Q_6_(P(DMAEMA-co-OEGMA)_1)_39_ aggregates remained well-defined upon heating. On the contrary, above 30 °C disaggregation phenomena took place in Q_12_(P(DMAEMA-co-OEGMA)_1)_50_ solution leading to collapse and intramolecularly self-folding into unimers (R_h_ = 1 nm) (Figure 6c). From 50 to 55 °C a slight growth in both populations is detected (Figure 6d), probably due to the presence of non-modified dimethylamino moieties, as a result of which, intramolecular interactions are demonstrated as temperature increases, thus additionally shielding the hydrophobic domain. Eventually, in this heterogeneous system (PDI = 0.4), the hydrophobic forces assigned to the long alkyl pendants did not prevail, and the phase transition was partially driven by the interaction with water molecules, hence supporting the assumption that these polyelectrolyte aggregates are conformationally loose. 

#### 3.3.4. Salt-Induced Responsive Behavior of Random Copolymers

Thermoresponsive polymer aggregation, such as PNIPAM, can be governed via the addition of salt into the aqueous solutions. In this regard, the salt described by the Hofmeister series can be utilized to trigger salting-out effects and thus reduce the hydration and solubility of the polymer. Several studies have successfully demonstrated the self-assembly and temperature-induced behavior of polyelectrolytes, including DMAEMA, in response to salt addition (such as NaCl) [44]. The random copolymers of the presented work were investigated upon NaCl (1 M) consecutively titration at pH = 7 and at ambient temperature.

**Figure 7:**
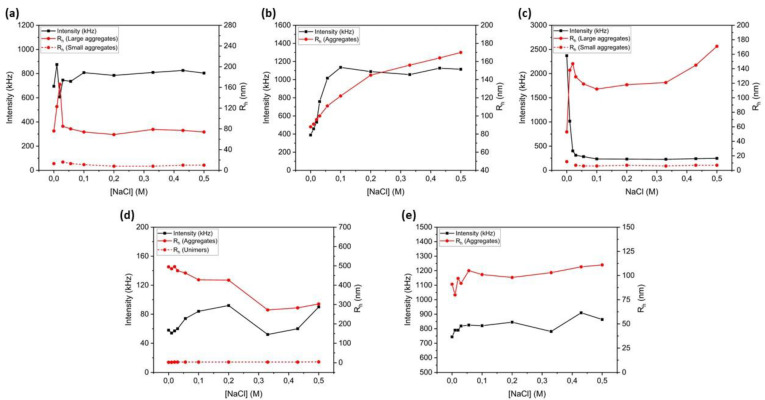
Salt-induced responsiveness of scattering intensity and hydrodynamic radius as determined from DLS measurements (at θ = 90° and pH = 7) for (**a**) P(DMAEMA-co-OEGMA)_1, (**b**) Q_1_(P(DMAEMA-co-OEGMA)_1)_39_, (**c**) Q_12_(P(DMAEMA-co-OEGMA)_1)_50_, (**d**) P(DMAEMA-co-OEGMA)_2 and (**e**) Q_1_(P(DMAEMA-co-OEGMA)_2)_50_ random copolymers.

The double-hydrophilic copolymers showed dissenting trends as far as their size and apparent mass variations are concerned. P(DMAEMA-co-OEGMA)_1 (Figure 7a) showed a substantial decrease of scattered intensity after the first salt addition (ca. 0.01 M NaCl), while the size was steeply decreased at ca. 0.02 M NaCl, provoking partial aggregate disintegration due to NaCl screening effects. Rather constant magnitudes of intensity and size were observed to further increase ionic strength (from 0.03 M and above, scattering intensity (I) and hydrodynamic radius (R_h_) attained a plateau). On the other hand, the mass of P(DMAEMA-co-OEGMA)_2 aggregates (Figure 7b) was found to fluctuate since aggregates became denser until 0.2 M NaCl (I = 92 kHz), further following a downward and upward trend, with a parallel decrease in overall size (R_h_ = 303 nm for aggregates and R_h_ = 4 nm for unimers at 0.5 M NaCl). The different response of double-hydrophilic copolymers to ionic strength variations is closely correlated with the contribution of parameters, including the interactions within the existing components (namely, intramolecular/intermolecular forces among polymers, polymer hydration, surface interactions with ions, ion linking or hydration), due to the complex random sequence and the composition of chain segments [46]. Regarding the polyelectrolytes, Q_1_(P(DMAEMA-co-OEGMA)_1)_100_ (Figure 7b), Q_1_(P(DMAEMA-co-OEGMA)_2)_100_ (Figure 7e) Q_6_(P(DMAEMA-co-OEGMA)_1)_39_ (Figure S3a), and Q_6_(P(DMAEMA-co-OEGMA)_2)_50_ (Figure S3b) aggregation phenomena took place from NaCl ~ 0.02 M and onwards as a result of salting-out effects. The apparent mass remained more or less steady, apart from Q_6_(P(DMAEMA-co-OEGMA)_2)_50_ for which it gradually rose. One could assume that in the latter polyelectrolyte solutions, polymer–polymer interactions predominate over polymer–water ones. Nonetheless, Q_12_(P(DMAEMA-co-OEGMA)_1)_50_ aggregates tended to swell after the first two additions of salt (the hydrodynamic radius increases steeply, coinciding with a decrease in mass). In consequent salt addition, the scattering intensity exhibited a plateau accompanied by a moderate increase in size (Figure 7e). This could be potentially attributed to the presence of several hydrophobic nanodomains within the aggregate's volume to prevent the collapse of QDMAEMA or to the water penetration (swelling) due to the aggregate loose conformation as a consequence of the increased salt concentration.

## 4. Conclusions

Stimuli-responsive P(DMAEMA-co-OEGMA) random copolymers of different compositions were efficiently synthesized via a single-step RAFT polymerization, as ascertained by SEC and ^1^H-NMR molecular characterization. Their chemical modification with methyl iodide, 1-iodohexane, and 1-iododecane, produced polyelectrolytes of different hydrophobic profiles and different overall charges. The polymer chain conformation and internal micropolarity were evidenced through fluorescence measurements whilst light scattering techniques provided a holistic picture related to the self-assembly behavior and response of double hydrophilic copolymers and their quaternized derivatives toward different stimuli. Each of the copolymer aqueous solutions exhibited noteworthy conformational and aggregation state changes, particularly in terms of aggregates’ mass, size, and density. Concerning the pH sensitivity, most of the copolymers were prone to further large aggregation (except for pH = 7 and 10 in the P(DMAEMA-co-OEGMA)_2 solution case, which showed the presence unimers), while the ethylene oxide units of OEGMA segments protected the hydrophobic inner aggregate domains. Both hydrophilic copolymers demonstrated temperature-responsive behavior, yet reversible, after the heating–cooling cycle, but P(DMAEMA-co-OEGMA)_2 had the most remarkable changes, judging from the amplified hydrophobicity at T = 55 °C. In addition, the modified derivatives displayed a moderate temperature and salt response due to the presence of alkyl chains that contribute different hydrophilicity/hydrophobicity profiles to the copolymers and can even mask their stimuli susceptibility. Eventually, the random segment sequence, the mass ratio of the segments, the hydrophilic/hydrophobic ratio, the length of attached alkyl pendant groups, the quaternization degree, and the presence of oligo ethylene side oxide units strongly influence how double-hydrophilic copolymers and their chemically modified analogs respond at different pH, temperature, and NaCl concentration, according to data derived from DLS and ELS measurements. Conclusively, the control of these parameters can efficiently determine the stimuli-responsive behavior of the present random copolymers and their subsequent utilization for protein and drug delivery purposes. 

## Figures and Tables

**Table 1:** Molecular characteristics of P(DMAEMA-co-OEGMA) double-hydrophilic random copolymers and their hydrophobically modified analogs.

Sample	M_w_^a^ (g∙mol^−1^) (×10^4^)	M_w_/M_n_^a^	% wt. DMAEMA ^b^	% wt. QDMAEMA ^b^	% wt. OEGMA ^b^
P(DMAEMA-co-OEGMA)_1	1.10	1.22	42	-	58
Q_1_(P(DMAEMA-co-OEGMA)_1)_100_	-	-	-	59 ^c^	41 ^c^
Q_6_(P(DMAEMA-co-OEGMA)_1)_39_	-	-	25	12	63
Q_12_(P(DMAEMA-co-OEGMA)_1)_50_	-	-	15 ^c^	41 ^c^	44 ^c^
P(DMAEMA-co-OEGMA)_2	2.28	1.26	76	-	24
Q_1_(P(DMAEMA-co-OEGMA)_2)_100_	-	-	-	72	28
Q_6_(P(DMAEMA-co-OEGMA)_2)_50_	-	-	56	31	13

^a^ Determined by SEC, ^b^ Determined by ^1^H-NMR, ^c^ calculated assuming 100% chemical modification with methyl iodide and 50% with 1-iodododecane.

**Table 2:** Internal micropolarity of Q(P(DMAEMA-co-OEGMA)_1) and Q(P(DMAEMA-co-OEGMA)_2) random copolymers from fluorescence measurements at different pHs.

Sample	pH	I_1_/I_3_
Q_1_(P(DMAEMA-co-OEGMA)_1)_100_	3	1.74
	7	1.68
	10	1.71
Q_6_(P(DMAEMA-co-OEGMA)_1)_39_	3	1.65
	7	1.64
	10	1.60
Q_12_(P(DMAEMA-co-OEGMA)_1)_50_	3	1.25
	7	1.21
	10	1.22
Q_1_(P(DMAEMA-co-OEGMA)_2)_100_	3	1.61
	7	1.64
	10	1.54
Q_6_(P(DMAEMA-co-OEGMA)_2)_50_	3	1.63
	7	1.58
	10	1.60

**Table 5:** Physicochemical features of double hydrophilic P(DMAEMA-co-OEGMA)_1 and P(DMAEMA-co-OEGMA)_2 random copolymers at neutral pH at two extreme temperatures.

Sample	Temperature (°C)	Intensity ^a^ (kHz)	R_h_^a^ (nm)	PDI ^a^
P(DMAEMA-co-OEGMA)_1	25	604	13/76	0.24
	55	732	66	0.31
P(DMAEMA-co-OEGMA)_2	25	66	2/86	0.59
	55	5320	692	0.13

^a^ Determined by DLS measurements at 90° and pH = 7. The Intensity values are determined with 1–2% error and the R_h_ ones with 5%; Note: The different R_h_ values at ambient temperature (25 °C) correspond to two different populations (unimers and aggregates, respectively) detected in the aqueous solution.

**Table 6:** Physicochemical characteristics of Q_6_(P(DMAEMA-co-OEGMA)_1)_39_ and Q_12_(P(DMAEMA-co-OEGMA)_1)_50_ random copolymers at neutral pH aqueous solution at two extreme temperatures.

Sample	Temperature (°C)	Intensity ^a^ (kHz)	R_h_ ^a^ (nm)	PDI ^a^
Q_6_(P(DMAEMA-co-OEGMA)_1)_39_	25	2019	71	0.16
	55	2039	54	0.03
Q_12_(P(DMAEMA-co-OEGMA)_1)_50_	25	266	11	0.41
	55	263	4 / 16	0.44

^a^ Determined by DLS measurements at 90° and pH = 7. The Intensity values are determined with 1–2% error and the R_h_ ones with 5%; Note: The different R_h_ values at 55 °C correspond to two different populations (unimers and aggregates, respectively) detected in the aqueous solution.

## Data Availability

Not applicable. No data have been created.

## Supplementary Materials

The following supporting information can be downloaded at: https://www.mdpi.com/article/10.3390/polym15061519/s1, Figure S1: Fluorescence spectra of (a) Q_6_(P(DMAEMA-co-OEGMA)_1)_39_ and (b) Q_12_(P(DMAEMA-co-OEGMA_1)_50_ modified random copolymers utilizing pyrene assay at different pH values and T = 25 °C; Figure S2: Distributions of hydrodynamic radius obtained from CONTIN analysis (at θ = 90° and pH 3, 7 and 10) for (a) Q_1_(P(DMAEMA-co-OEGMA)_1)_100_, (b) Q_6_(P(DMAEMA-co-OEGMA)_1)_39_, (c) Q_12_(P(DMAEMA-co-OEGMA)_1)_50_, (d) Q_1_(P(DMAEMA-co-OEGMA)_2)_100_ and (e) Q_6_(P(DMAEMA-co-OEGMA)_1)_50_.; Figure S3: Variation of the scattering intensity and hydrodynamic radius (R_h_) as a function of ionic strength for (a) Q_6_(P(DMAEMA-co-OEGMA)_1)_50_ and (b) Q_6_(P(DMAEMA-co-OEGMA)_2)_50_ modified random copolymers.
