# Supplementary material for: Stimuli-Responsive Self-Assembly of Poly(2-(Dimethylamino)ethyl Methacrylate-co-(oligo ethylene glycol)methacrylate) Random Copolymers and Their Modified Derivatives
Source: Polymers (Basel). 2023 Mar 19;15(6):1519. doi: 10.3390/polym15061519 (PMC10059824; doi:10.3390/polym15061519)
Supplement: Supplementary file 1 [file polymers-15-01519-s001.zip › polymers-2273171-supplementary.pdf]

Supplementary Materials

Stimuli-Responsive Self-Assembly of Poly(2-(dimethylamino)ethyl methacrylate-co-(oligo ethylene glycol)methacrylate) Random Copolymers and Their Modified Derivatives

Antiopi Vardaxi [1,2] and Stergios Pispas [1*]

[1] Theoretical and Physical Chemistry Institute, National Hellenic Research Foundation, 48 Vassileos Constantinou Avenue, 11635, Athens, Greece; ada.vardaxi@gmail.com,

[2] Department of Chemistry, National and Kapodistrian University of Athens (NKUA), 15784, Athens, Greece

* Correspondence: pispas@eie.gr ; Tel.: +30-210-7273824

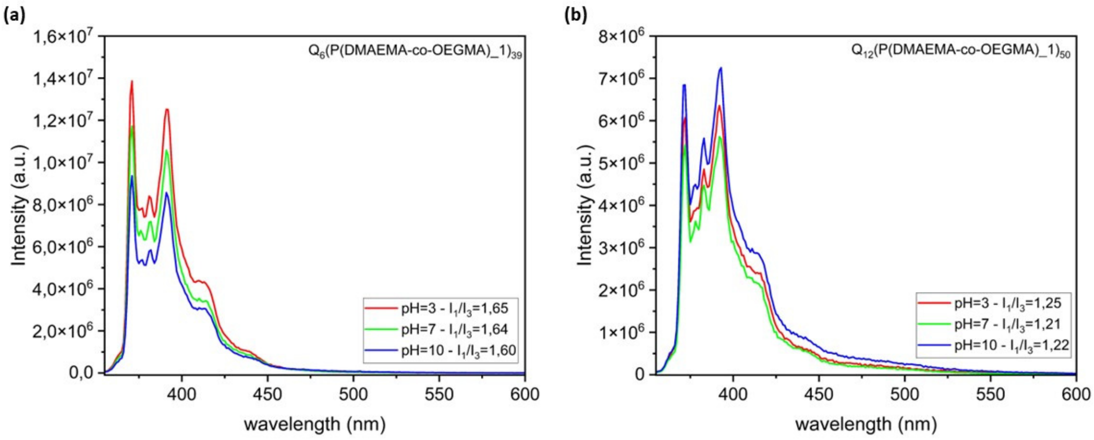

Figure S1: Fluorescence spectra of (a) Q_6(P(DMAEMA-co-OEGMA)_1)$_{39}$ and (b) Q_{12}(P(DMAEMA-co-OEGMA_1)$_{50}$ modified random copolymers utilizing pyrene assay at different pH values and T = 25 ^{o}C.

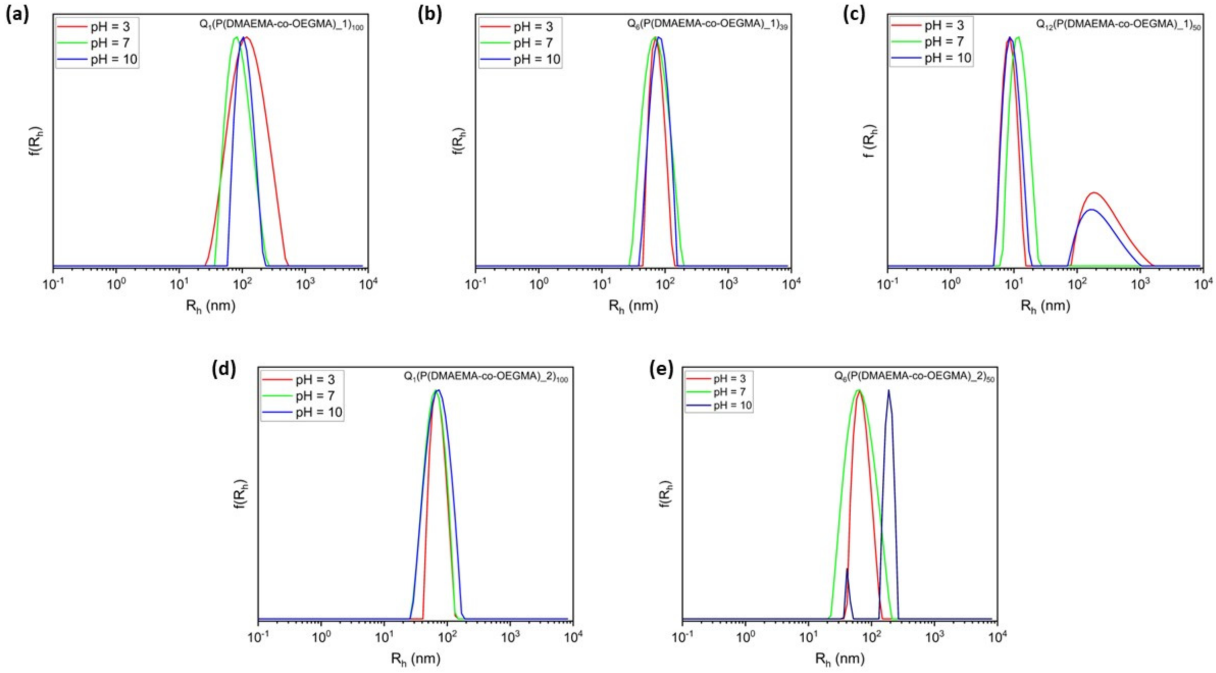

Figure S2: Distributions of hydrodynamic radius obtained from CONTIN analysis (at θ = 90° and pH 3, 7 and 10) for (a) Q_1(P(DMAEMA-co-OEGMA)_1)$_{100}$, (b) Q_6(P(DMAEMA-co-OEGMA)_1)$_{39}$, (c) Q_{12}(P(DMAEMA-co-OEGMA)_1)$_{50}$, (d) Q_1(P(DMAEMA-co-OEGMA)_2)$_{100}$ and (e) Q_6(P(DMAEMA-co-OEGMA)_1)$_{50}$.

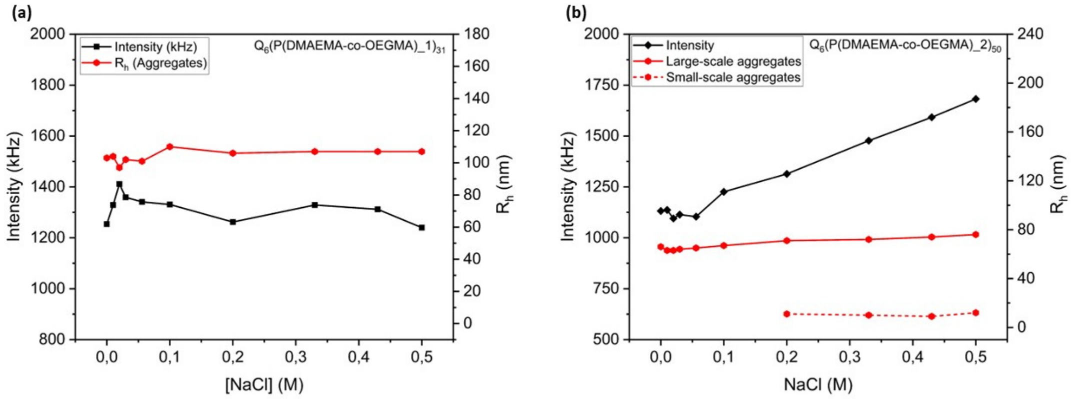

Figure S3: Variation of the scattering intensity and hydrodynamic radius (R_h) as a function of ionic strength for (a) Q_6(P(DMAEMA-co-OEGMA)_1)$_{50}$ and (b) Q_6(P(DMAEMA-co-OEGMA)_2)$_{50}$ modified random copolymers.
